# Frequency of rare recessive mutations in unexplained late onset cerebellar ataxia

**DOI:** 10.1007/s00415-015-7772-x

**Published:** 2015-05-16

**Authors:** M. J. Keogh, H. Steele, K. Douroudis, A. Pyle, J. Duff, R. Hussain, T. Smertenko, H. Griffin, M. Santibanez-Koref, R. Horvath, P. F. Chinnery

**Affiliations:** Institute of Genetic Medicine, Newcastle University, Newcastle upon Tyne, NE1 3BZ UK; Department of Neurology, Royal Victoria Infirmary, Newcastle upon Tyne, NE1 4LP UK

**Keywords:** Ataxia, Whole exome sequencing, Next generation sequencing, Diagnosis

## Abstract

Sporadic late onset cerebellar ataxia is a well-described clinical presentation with a broad differential diagnosis that adult neurologists should be familiar with. However, despite extensive clinical investigations, an acquired cause is identified in only a minority of cases. Thereafter, an underlying genetic basis is often considered, even in those without a family history. Here we apply whole exome sequencing to a cohort of 12 patients with late onset cerebellar ataxia. We show that 33 % of ‘idiopathic’ cases harbor compound heterozygous mutations in known ataxia genes, including genes not included on multi-gene panels, or primarily associated with an ataxic presentation.

**Electronic supplementary material:**

The online version of this article (doi:10.1007/s00415-015-7772-x) contains supplementary material, which is available to authorized users.

## Introduction

Adult onset cerebellar ataxia poses a considerable diagnostic challenge. Initial investigations focus on detecting degenerative, toxic, structural and inflammatory etiologies which together underlie around a third of cases [1]. Thereafter, molecular investigations for a monogenic basis of disease are often undertaken despite 80 % of patients having no relevant family history [2].

Current molecular investigations for sporadic cases echo that of familial forms, beginning with testing for trinucleotide repeat disorders, such as the spinocerebellar ataxias (SCA1, 2, 3, 6, 7 and 17), dentatorubral pallidoluysian atrophy (DRPLA) and Friedreich’s ataxia (FDR) in most centres [1]. However, this approach fails to identify a molecular diagnosis in 87–98 % of late onset sporadic cases [1, 3], and subsequent investigations are undertaken on a gene-by-gene basis, often at considerable time and expense.

The difficulty in establishing monogenic forms of disease using this approach is increasingly challenging given that at least 60 causative ataxia genes are reported [4]. Recent studies have therefore utilized next generation sequencing focusing on infantile or juvenile onset cases [5], or adult onset ataxia with a demonstrable family history [4]. Only two studies have described sub-sets of patients with sporadic onset adult disease, despite it being a major form of ataxia, and suggested that a molecular diagnosis can be reached in ~10 % of cases [4, 6]. Given this, we applied whole exome sequencing to a cohort of individuals with sporadic late onset ataxia.

## Methods

Unrelated individuals with sporadic ataxia beginning at 30 years of age or over were identified from routine referrals to our regional neurogenetic service, in Newcastle upon Tyne, England.

Acquired causes of ataxia were excluded and all participants had negative genetic testing for SCA 1, 2, 3, 6, 7, 17, *DRPLA* and Friedreich’s Ataxia (FA). In addition, all adult males had negative *FMR1* testing.

Blood genomic DNA was fragmented, exome enriched and sequenced (Nextera Rapid Exome Capture 37 Mb and HiSeq 2000, 100 bp paired-end reads). In-house bioinformatic analysis included alignment to UCSC hg19, using BWA as aligner and GATK to detect SNV and INDELS across all samples using standard filtering parameters according to GATK Best Practise Recommendations [7] (see supplementary methods). Further analysis was performed on variants with a minor allele frequency <0.005 in several reference databases and 302 unrelated in-house controls (see supplementary methods). Rare heterozygous, homozygous and compound heterozygous variants were defined, and protein altering and/or putative ‘disease causing’ mutations as predicted by at least three out of four software programmes were included. Pathogenicity was defined in accordance with American College of Medical Genetic guidelines (see supplementary methods). Genes known or suggested to cause ataxia as a primary or secondary phenotype in humans from two suggested clinical panels [4, 8] together with additional genes in which ataxia may result as part of the phenotype (list-supplementary methods) were assessed for variants according to the above criteria, and confirmed by Sanger sequencing (supplementary methods).

Variants were defined using a priori criteria: (1) confirmed pathogenic: dominant disorders*—*variant previously shown to cause ataxia in humans; recessive disorders*—*either 2 variants previously shown to cause ataxia in humans; or 1 pathogenic variant with a second variant predicted to affect protein function by at least 3 of 4 prediction algorithms (SIFT, Polyphen2, Mutation Taster, LRT), or through frameshift or truncation. (2) Probable pathogenic: dominant and recessive disorders—variants in known genes causing ataxia in humans and predicted to affect protein function by at least three of four prediction algorithms; (3) uncertain significance: dominant and recessive disorders—variants predicted to affect protein function with weak evidence that gene alteration causes ataxia in humans.

The study was granted ethical approval from a Research Ethics Committee based in the North of England.

## Results

### Population

Twelve Caucasian individuals of British origin (5 male) with no known consanguinity were included (Table 1). Mean age at disease onset was 46.7 years (SD 11; range 30–70 years). Mean disease duration was 16.6 years (SD 6.9; range 6–30 years). For one patient, the disease duration fell within the range expected for multi-system atrophy [9]. This patient had a normal DaTscan and autonomic function tests. Three individuals had CSF examination with negative oligoclonal bands. Five had nerve conduction studies; two of which were abnormal. Detailed clinical features and the results of clinical investigations are shown in Table 1.

**Table 1:** Clinical features of the 12 patients in the cohort

Patient no., sex	Age (years)	Age onset (years)	Disease duration (years)	Presenting symptom	Gait ataxia	Limb ataxia	Ocular signs	Additional neurological features	Other features	MRI	LP	NCS/EMG	Other investigations	Muscle biopsy	Other negative molecular investigations
1, F	63	40	23	Slowly progressive midline and appendicular ataxic syndrome	+++	++	Early CPEO Dysmetric pursuit Broken saccades	Dysphagia, spastic bladder Lower limb spasticity	None	CA	Normal −OCB	Bilateral CTS (CTS study only)		Normal IHC No mtDNA deletions	*FMR1*
2, F	47	30	17	Slowly progressive spastic ataxic syndrome	++ (Fr)	+	CPEO Temporal optic disc pallor Jerky pursuit Hypometric saccades	Spastic lower limbs Brisk reflexes	None	Mild CA	Normal −OCB	ND		Mild fibre size variation Low Q10	Nil
3, F	57	45	12	Ataxia developed aged 45	+++	++	Slow saccades	Epilepsy aged 7	None	CA and parieto-occipital atrophy	ND	Normal		ND	*FMR1*
4, F	63	40	23	Slowly progressive midline cerebellar ataxia	++	+	GEN	TLE with ongoing infrequent focal seizures, no treatment	Cataracts (age 62)	CA	ND	ND		ND	*POLG* *MT*-*ATP6 &* 8
5, F	55	35	20	Slowly progressive spastic ataxic syndrome	+++ (WhC)	++	Jerky pursuit GEN Hypometric saccades	Neurogenic bladder Spastic ataxic gait Brisk reflexes Positive Babinski	None	CA	ND	ND		ND	*SPG7*
6, F	76	70	6	Progressive midline and appendicular ataxia	+++	++	GEN Up and down beat nystagmus	Orthostatic tremor Brisk reflexes	None	Mild CA	ND	ND	−DaT	Normal IHC No mtDNA deletions	*MT*-*ATP 6 & 8*
7, M	71	60	11	Slowly progressive midline ataxia	+	−	Jerky ocular pursuit GEN	None	None	CA	ND	Normal		ND	*SPG7* *MT*-*ATP 6 & 8*
8, M	58	50	8	Midline ataxia	++	+	RAPD OA Jerky pursuit GEN	Congenital hearing loss Early dysphagia Areflexia	None	CA	ND	SAN		Normal IHC No mtDNA deletions Normal Q10	*POLG* *WFS1* *OPA1* *MT*-*ATP 6 & 8*
9, M	70	40	30	Pure midline ataxia	+ (stick)	−	None	None	None	CA	ND	ND		Normal IHC Normal RCE	SCA12 mt.DNA LR-PCR
10, M	59	44	15	Pure midline ataxia	+++	+	None	Prominent dysarthria, choking Brisk reflexes	None	CA	ND	ND		Normal Q10	*SPG7* SCA8 SCA12
11, F	65	47	12	Pure midline ataxia	+++ (WhC)	+	Oscillopsia Jerky pursuits Horizontal nystagmus Hypometric saccades	Dorsal root ganglionopathy Neurogenic bladder Distal wasting and weakness Areflexia	Cataract, diabetes and short stature	Mild CA; high signal C3, 4 posterior columns; thin cord	−OCB	DRG		ND	*POLG* *SPG7* *POLG2* *PEO1* *ANT1* mt.DNA LR-PCR
12, M	83	60	23	Midline ataxia Early alcohol sensitivity	++ (stick)	+	Jerky pursuit Coarse phasic nystagmus Normal saccades	None	None	Mild CA	ND	ND		Patient declined	Nil

Presence or absence of symptoms are indicated by + or − symbol, respectively

*AFTs* autonomic function tests, *CA* Cerebellar atrophy, *CPEO* chronic progressive external ophthalmoplegia, *CTS* carpal tunnel syndrome, *CVD* cerebrovascular disease, *DRG* dorsal root ganglionopathy, *Fr* Frame, *GEN* gaze evoked nystagmus, *IHC* immunohistochemistry, *ND* not done, *OA* optic atrophy, *OCB* oligoclonal bands, *PV* periventricular, *RAPD* relative afferent pupillary defect, *RCE* respiratory chain enzyme, *SVD* small vessel disease, *TLE* temporal lobe epilepsy, *WhC* wheelchair, *WM* white matter

### Diagnosis

We identified previously described pathogenic mutations in four of the 12 (33 %) patients in our cohort. All were present on confirmatory Sanger sequencing. No probable pathogenic variants were identified and variants of uncertain significance were found in an additional two cases (17 %). Findings are summarised in Table 2.

**Table 2:** Genetic variants of interest identified in the 12 patients

Pathogenic variants											
Pt	Gene	Model	Exome seq identified variant (1)	rs#	MAF variant (1)		Exome seq identified variant (2)	rs#	MAF variant (2)		Variant pathogenicity prediction
					ESP6500	1000 g			ESP6500	1000 g	
1	*SPG7*	AR	c.1529C>T p. Ala510Val	rs61755320	0.003463	0.0014	c. 1053dupC p. Gly352fs	NA	0	0	(1) D:D:D:D (2) NA
2	*SPG7*	AR	c.1529C>T p. Ala510Val	rs61755320	0.003463	0.0014	c.233T>A p. Leu78*	rs121918358	0.000077	0	(1) D:D:D:D (2) Pathogenic
3	*ANO10*	AR	c.1843G>A p. Asp615Asn	rs138000380	0.000231	0.0005	c. 132_133insT p. Asp45fs	NA	0	0	(1) D:D:D:P (2) NA
4	*SYNE1*	AR	c.9148C>G p. Leu3050Val	rs117360770	0.002307	0.0018	c.1762delC p. Leu588fs	NA	0.003435	0	(1) D:D:D:D (2) NA

Variants of uncertain significance											
5	*SLC33A1*	AD	c.433G>A p. Gly145Ser	rs138283229	0.002461	0.0009	NA		NA	NA	D:D:D:D
6	*PLEKHG4*	AD	c.2251G>A p. Asp751Asn	NA	0.000077	0	NA		NA	NA	D:D:N:D

Confirmed pathogenic: dominant disorders—variant previously shown to cause ataxia in humans; recessive disorders—either 2 variants previously shown to cause ataxia in humans; or 1 pathogenic variant with a second variant predicted to affect protein function by at least 3 of 4 prediction algorithms (SIFT, Polyphen2, Mutation Taster, LRT), through frameshift or truncation. Variants of uncertain significance: dominant and recessive disorders—variants predicted to affect protein function with weak evidence that gene alteration causes ataxia in humans

*D* pathogenic or deleterious, *P* polymorphism, *NA* not applicable *N* neutral (frameshift mutations considered pathogenic)

## Discussion

We identified confirmed or probable pathogenic variants causing sporadic late onset ataxia in four patients (33 %) in our cohort. These findings are comparable to childhood/adolescent ataxia using targeted sequencing panels (40 %) [4] and whole exome sequencing (27 %) [5]. They are also significantly higher than previous data for adult onset cases using either panels or whole exome (both ~10 %) [4, 6].

We detected pathogenic variants in *SPG7, SYNE1 and ANO10* (previously published by Balreira et al. [10]). Fogel et al. [6] also identified pathogenic variants in these genes (*SPG7* (*n* = 2), *SYNE1* (*n* = 3) and *ANO10* (*n* = 1). The clinical features of these patients appear relatively homogenous between their and our study, with pure cerebellar ataxia beginning above the age of 40 for *ANO10* and *SYNE1* cases, and a more heterogeneous age of onset (<20–50) with additional neurological features including spasticity and a polyneuropathy in *SPG7* cases [6]. Therefore, pathogenic mutations in these genes appear to be an important and frequently identified cause of late onset sporadic ataxia.

We used whole exome sequencing (WES) rather than targeted next generation ‘panels’, and it remains a contentious issue as to which is more appropriate in the investigation of neurogenetic disorders. WES enables greater genome coverage, and hence detection of pathogenic mutations in genes not considered as having ataxia as a primary phenotype. Our results highlight this as *SPG7* was not covered by one ataxia panel [4], *SYNE1* by another [8], and *ANO10* was not included in either panel. WES however, may result in detection of unexpected findings such as pathogenic mutations predisposing to cancer or neurodegenerative disease, which must be considered and included in appropriate consent procedures. It must also be noted that neither WES nor targeted panels are appropriate to screen for genomic rearrangements or trinucleotide repeat sequences.

Determining pathogenicity can be challenging for heterozygous variants without a family history of disease and additional living family relatives for segregation analysis. In our cohort, we found heterozygous variants in *SLC33A1* and *PLEKHG4* in single cases (Table 2). Heterozygous mutations in *SLC33A1* have been associated with spastic paraplegia (SPG42) with ataxia in a single family, and likewise, missense mutations in *PLEKHG4* have been implicated in dominant late onset forms of spinocerebellar ataxia in Japanese individuals. Despite the rarity and putative pathogenicity of the variants in our patients, the lack of data to test segregation makes attributing pathogenicity difficult. As NGS begins to develop larger variant datasets in rare diseases it is vital to share such data through collaborative projects which may aid pathogenicity confirmation through the identification of the same or related variants in unrelated families with a similar phenotype.

In conclusion, we have demonstrated that application of WES to a cohort of unrelated individuals following exclusion of common trinucleotide repeat disorders establishes a molecular cause of disease in a third of cases. These findings have significant implications for clinical practise.

## Electronic supplementary material

Supplementary material 1 (DOCX 48 kb)
