# Supplementary material for: Frequency of rare recessive mutations in unexplained late onset cerebellar ataxia
Source: J Neurol. 2015 May 16;262(8):1822–7. doi: 10.1007/s00415-015-7772-x (PMC4539354; doi:10.1007/s00415-015-7772-x)
Supplement: Supplementary file 1 — Supplementary material 1 (DOCX 48 kb) [file 415_2015_7772_MOESM1_ESM.docx]

**Supplementary information**

Supplementary methods

Blood genomic DNA was fragmented, enriched and seqeunced as described in the main text. Further analysis was performed on variants with a minor allele frequency <0.005 in several databases: dbSNP137, 1000 genomes (April 2012 data release), the National Heart, Lung and Blood Institute (NIH, Bethesda, MD) Exome Sequencing Project (ESP) 6500 exomes, and 302 unrelated in-house controls without ataxia as a primary or secondary phenotype. Rare heterozygous, homozygous and compound heterozygous variants were defined, and protein altering variants along with their functional annotation, were identified using ANNOVAR [^3^](#_ENREF_3). Subsequently variants were analyzed by MutationTaster [^4^](#_ENREF_4), SIFT [^5^](#_ENREF_5), Polyphen2 [^6^](#_ENREF_6) and LRT [^7^](#_ENREF_7) to determine functional impact. Variants which were predicted by at least 3 of these 4 tools to have a putative AR pathogenic or deleterious function were included. In addition, all variants met the criteria for reporting pathogenicity as determined by the American College of Medical Genetics [^8^](#_ENREF_8). Subsequently variants meeting this criteria (Table 1 in paper) were confirmed by Sanger sequencing using custom-designed primers (<http://frodo.wi.mit.edu>) (ABI BigDye v3.1 3130xl Genetic Analyzer, Life Technologies, Warrington, UK) as listed below. Comparative genomic hybridization was also performed in presumed recessive cases where a single likely pathogenic allele was found in a strong candidate gene, but showed no evidence of large scale deletions or rearrangements in any case, nor evidence of short deletions or rearrangements in association with pathogenic or putative ARly pathogenic SNVs (data not shown).

**Supplementary tables**

| Gene | Inheritance | Paper / panel | | |
| --- | --- | --- | --- | --- |
|  |  | Nemeth et al [^9^](#_ENREF_9) | Sheffield Diagnostic panel [^10^](#_ENREF_10) | Additional genes (in-house) |
| *ATXN1* | AD | ✓ |  |  |
| *ATXN2* | AD | ✓ |  |  |
| *ATXN3* | AD | ✓ |  |  |
| *PLEKHG4* | AD | ✓ |  |  |
| *SPTBN2* | AD | ✓ | ✓ |  |
| *CACNA1A* | AD | ✓ | ✓ |  |
| *ATXN7* | AD | ✓ |  |  |
| *ATXN8OS* | AD | ✓ |  |  |
| *ATXN10* | AD | ✓ |  |  |
| *TTBK2* | AD | ✓ | ✓ |  |
| *KCNC3* | AD | ✓ | ✓ |  |
| *PRKCG* | AD | ✓ | ✓ |  |
| *ITPR1* | AD | ✓ | ✓ |  |
| *TBP* | AD | ✓ |  |  |
| *FGF14* | AD | ✓ | ✓ |  |
| *AFG3L2* | AD | ✓ | ✓ |  |
| *BEAN1* | AD | ✓ |  |  |
| *KCNA1* | AD | ✓ | ✓ |  |
| *CACNB4* | AD | ✓ | ✓ |  |
| *SLC1A3* | AD | ✓ | ✓ |  |
| *ATN1* | AD | ✓ |  |  |
| *PPP2R2B* | AD | ✓ |  |  |
| *APTX* | AR | ✓ | ✓ |  |
| *SACS* | AR | ✓ | ✓ |  |
| *ABCB7* | AR | ✓ | ✓ |  |
| *ATM* | AR | ✓ | ✓ |  |
| *MRE11A* | AR | ✓ |  |  |
| *TTPA* | AR | ✓ | ✓ |  |
| *ATCAY* | AR | ✓ |  |  |
| *FXN* | AR | ✓ | ✓ |  |
| *SIL1* | AR | ✓ | ✓ |  |
| *TDP1* | AR | ✓ |  |  |
| *CABC1* | AR | ✓ |  |  |
| *SETX* | AR | ✓ | ✓ |  |
| *SYNE1* | AR | ✓ |  |  |
| *MARS2* | AR | ✓ |  |  |
| *GRM1* | AR | ✓ |  |  |
| *KIAA0226* | AR | ✓ |  |  |
| *DARS2* | AR | ✓ |  |  |
| *KCNJ10* | AR | ✓ |  |  |
| *AAAS* | AR | ✓ |  |  |
| *VLDLR* | AR | ✓ |  |  |
| *PRPS1* | X-linked | ✓ |  |  |
| *GAN* | AD | ✓ |  |  |
| *SLC6A19* | AR | ✓ |  |  |
| *TINF2* | AD | ✓ |  |  |
| *PSAP* | AR | ✓ |  |  |
| *SCN1A* | AD | ✓ | ✓ |  |
| *AARS2* | AR | ✓ |  |  |
| *CARS* | putative AR | ✓ |  |  |
| *CARS2* | putative AR | ✓ |  |  |
| *DARS* | AR | ✓ |  |  |
| *DARS2* | AR | ✓ |  |  |
| *EARS2* | AR | ✓ |  |  |
| *EPRS* | putative AR | ✓ |  |  |
| *FARS2* | AR | ✓ |  |  |
| *FARSA* | putative AR | ✓ |  |  |
| *FARSB* | putative AR | ✓ |  |  |
| *GARS* | AD | ✓ |  |  |
| *HARS* | AR | ✓ |  |  |
| *HARS2* | AR | ✓ |  |  |
| *IARS* | putative AR | ✓ |  |  |
| *IARS2* | AR | ✓ |  |  |
| *KARS* | AR | ✓ |  |  |
| *LARS* | AR | ✓ |  |  |
| *LARS2* | AR | ✓ |  |  |
| *MARS* | AR | ✓ |  |  |
| *NARS* | putative AR | ✓ |  |  |
| *NARS2* | putative AR | ✓ |  |  |
| *PARS2* | putative AR | ✓ |  |  |
| *QARS* | AR | ✓ |  |  |
| *RARS* | AR | ✓ |  |  |
| *RARS2* | AR | ✓ |  |  |
| *SARS* | putative AR | ✓ |  |  |
| *SARS2* | AR | ✓ |  |  |
| *TARS* | putative AR | ✓ |  |  |
| *TARS2* | AR | ✓ |  |  |
| *VARS* | putative AR | ✓ |  |  |
| *VARS2* | AR | ✓ |  |  |
| *WARS* | putative AR | ✓ |  |  |
| *WARS2* | putative AR | ✓ |  |  |
| *YARS* | AD | ✓ |  |  |
| *YARS2* | AR | ✓ |  |  |
| *AHI1* | AR | ✓ |  |  |
| *ARL13B* | AR | ✓ |  |  |
| *CC2D2A* | AR | ✓ |  |  |
| *CEP290* | AR | ✓ |  |  |
| *INPP5E* | AR | ✓ |  |  |
| *NPHP1* | AR | ✓ |  |  |
| *OFD1* | X-linked | ✓ |  |  |
| *RPGRIP1L* | AR | ✓ |  |  |
| *TMEM216* | AR | ✓ |  |  |
| *TMEM67* | AR | ✓ |  |  |
| *ATP1A2* | AD |  | ✓ |  |
| *ATP1A3* | AD |  | ✓ |  |
| *ATP7B* | AR |  | ✓ |  |
| *C10orf2* | AR/AD |  | ✓ |  |
| *CACNB4* | AD |  | ✓ |  |
| *CYP27A1* | AR |  | ✓ |  |
| *EEF2* | AD |  | ✓ |  |
| *FTL* | AD |  | ✓ |  |
| *FXN* | AR |  | ✓ |  |
| *GBA* | AR/AD |  | ✓ |  |
| *GBA2* | AR |  | ✓ |  |
| *IFRD1* | AD |  | ✓ |  |
| *KCND3* | AD |  | ✓ |  |
| *MTPAP* | AR |  | ✓ |  |
| *PDYN* | AD |  | ✓ |  |
| *PRRT2* | AD |  | ✓ |  |
| *SETX* | AR |  | ✓ |  |
| *SLC16A2* | X-Linked |  | ✓ |  |
| *SLC2A1* | AD |  | ✓ |  |
| *SPG7* | AR |  | ✓ |  |
| *TGM6* | AD |  | ✓ |  |
| *VAMP1* | AD |  | ✓ |  |
| *ABCB7* | X-linked |  |  | ✓ |
| *ANO10* | AR |  |  | ✓ |
| *ATP8A2* | AR |  |  | ✓ |
| *COQ2* | AD |  |  | ✓ |
| *COQ9* | AR |  |  | ✓ |
| *NOP56* | AD |  |  | ✓ |
| *PDSS1* | AR |  |  | ✓ |
| *PDSS2* | AR |  |  | ✓ |
| *PEX7* | AR |  |  | ✓ |
| *PHYH* | AR |  |  | ✓ |
| *SCN8A* | AR |  |  | ✓ |
| *SYT14* | AR |  |  | ✓ |

**Supplementary table 1:** A table of genes analyzed in the study. Genes attributed to cause ataxia as a primary or secondary phenotype in humans were included from a published clinical study (Nemeth et al) and clinically available diagnostic panel (Sheffield – NHS UK) for ataxia together with additional genes which have been associated with ataxia but were not included with either panel (Additional Genes – in house). AR: autosomal recessive; AD: autosomal dominant;

| Patient_ID | Total_target_bases | Mean_target_base_coverage | Number CCDS bases covered 20-fold | % CCDS bases covered 20-fold | Number CCDS bases covered 10-fold | % CCDS bases covered 10-fold | Number CCDS bases covered 5-fold | % CCDS bases covered 5-fold | Number CCDS bases covered 1-fold | % CCDS bases covered 1-fold |
| --- | --- | --- | --- | --- | --- | --- | --- | --- | --- | --- |
| 1 | 31935069 | 50.8 | 25133972 | 78.7 | 29232001 | 91.5 | 30838092 | 96.6 | 31749555 | 99.4 |
| 2 | 31935069 | 80.5 | 29413469 | 92.1 | 30486628 | 95.5 | 30910191 | 96.8 | 31363939 | 98.2 |
| 3 | 31935069 | 71.7 | 28999803 | 90.8 | 30339864 | 95.0 | 30836709 | 96.6 | 31313756 | 98.1 |
| 4 | 31935069 | 51.9 | 25198617 | 78.9 | 29282189 | 91.7 | 30866092 | 96.7 | 31744017 | 99.4 |
| 5 | 31935069 | 47.5 | 24427407 | 76.5 | 28924723 | 90.6 | 30712439 | 96.2 | 31725909 | 99.4 |
| 6 | 31935069 | 46.4 | 24036923 | 75.3 | 28735381 | 90.0 | 30648722 | 96.0 | 31722518 | 99.3 |
| 7 | 31935069 | 55.9 | 25961966 | 81.3 | 29686530 | 93.0 | 31066887 | 97.3 | 31800775 | 99.6 |
| 8 | 31935069 | 56.9 | 26055133 | 81.6 | 29669893 | 92.9 | 31058162 | 97.3 | 31814076 | 99.6 |
| 9 | 31935069 | 47.3 | 24473131 | 76.6 | 29031772 | 90.9 | 30809911 | 96.5 | 31775198 | 99.5 |
| 10 | 31935069 | 59.9 | 26164544 | 81.9 | 29591174 | 92.7 | 30990851 | 97.0 | 31794752 | 99.6 |
| 11 | 31935069 | 60.8 | 27151336 | 85.0 | 30242676 | 94.7 | 31291169 | 98.0 | 31817250 | 99.6 |
| 12 | 31935069 | 57.2 | 26143666 | 81.9 | 29711610 | 93.0 | 31077954 | 97.3 | 31814848 | 99.6 |

**Supplementary table 2:** Coverage data for all CCDS bases across the whole exome for all 12 patients in the study (hg19 chromosome build).

| Patient ID | Total CCDS bases (Ataxia genes) | Mean per base Depth | Number bases covered 20-fold | % bases covered 20-fold | Number bases covered 10-fold | % bases covered 10-fold | Number bases covered 5-fold | % bases covered 5-fold | Number bases covered 1-fold | % bases covered 1-fold |
| --- | --- | --- | --- | --- | --- | --- | --- | --- | --- | --- |
| 1 | 325483 | 74.5 | 292575 | 91.4 | 314685 | 97.5 | 321141 | 99.0 | 324633 | 99.8 |
| 2 | 325483 | 71.9 | 309429 | 95.4 | 317111 | 97.5 | 319940 | 98.3 | 322700 | 99.0 |
| 3 | 325483 | 64.5 | 307173 | 95.0 | 315851 | 97.3 | 319352 | 98.1 | 321601 | 98.8 |
| 4 | 325483 | 75.7 | 293987 | 91.5 | 314562 | 97.3 | 320705 | 99.0 | 324702 | 99.8 |
| 5 | 325483 | 69.5 | 287401 | 90.1 | 312163 | 97.0 | 320107 | 98.9 | 324753 | 99.9 |
| 6 | 325483 | 68.2 | 286359 | 89.7 | 311771 | 96.8 | 320053 | 98.8 | 324746 | 99.8 |
| 7 | 325483 | 81.3 | 299394 | 93.2 | 316575 | 98.0 | 321890 | 99.2 | 324750 | 99.8 |
| 8 | 325483 | 83.4 | 298195 | 93.2 | 315454 | 97.8 | 321272 | 99.2 | 324923 | 99.9 |
| 9 | 325483 | 68.7 | 289741 | 90.6 | 312539 | 97.1 | 320943 | 99.0 | 324810 | 99.8 |
| 10 | 325483 | 88.8 | 298269 | 93.3 | 315106 | 97.6 | 321243 | 99.0 | 324861 | 99.9 |
| 11 | 325483 | 85.3 | 304636 | 94.4 | 318829 | 98.4 | 322824 | 99.4 | 325140 | 99.9 |
| 12 | 325483 | 83.5 | 299543 | 93.3 | 316386 | 98.0 | 321647 | 99.2 | 324620 | 99.8 |

**Supplementary table 3**: Coverage data for all CCDS bases in genes causing ataxia in humans and assessed in this study (full list see Supplementary table 1) for all 12 patients in the study (hg19 chromosome build).

| **Gene** | **ENGS gene ID** | **Ch Position** | **cDNA change and protein alteration** | **rs number** | **Forward Primer** | **Reverse Primer** |
| --- | --- | --- | --- | --- | --- | --- |
| *SPG7* | ENSG00000197912 | 89613145 | c. C1529T  p. Ala510Val | rs61755320 | AGGCATGATGGCACACACT | CTGCCCCACATACACATCAG |
| *SPG7* | ENSG00000197912 | 89598370 | c. 1053dupC p. Gly352fs | NA | TTGAACCCAGGAGTTTGAGG | TCCCTTTCAGTCCAGACACC |
| *SPG7* | ENSG00000197912 | 89613145 | c. C1529T  p. Ala510Val | rs61755320 | AGGCATGATGGCACACACT | CTGCCCCACATACACATCAG |
| *SPG7* | ENSG00000197912 | 89576947 | c.T233A  p.Leu78X | rs121918358 | GCTTTGACCTATTGCTCAGACT | GCGAGATCCCGTCTCTAAAA |
| *ANO10* | ENSG00000160746 | 43474174 | c.G1843A  p.Asp615Asn | rs138000380 | GGCCTGCTTGGTCTTTGATAC | TCCTGAACTGGAGTCCTCTG |
| *ANO10* | ENSG00000160746 | 43647212 | c.132_133insT  p.Asp45fs | NA | TGCTTTTATCTTGGAAGCCAG | GGGAGGCTGAGCATACAGTG |
| *SYNE1* | ENSG00000131018 | 152697692 | c. C9148G p. Leu3050Val | rs117360770 | ACACCCTTACCTGTATTTTCTGA | GAGACCTGAGTACCTACAGTGG |
| *SYNE1* | ENSG00000131018 | 152774686 | c. 1762delC  p. Leu78* | NA | AAATGTTCTTGCACCCTGGA | GCCTTTTCGTCTGTCCAACA |
| *SLC33A1* | ENSG00000169359 | 155546124 | c. G433A  p. Gly145Ser | rs138283229 | AATTACCTTGCTAGAATGTCCAGTAGC | CCACCTCAGCCTTGTGAGTAGAT |
| *PLEKHG4* | ENSG00000196155 | 67319264 | c.G2251A  p.Asp751Asn | NA | CAGCTCTGACCCCAGGAG | TGTGGCAGAACGAGCTGATA |

**Supplementary table 4.** Details of primers used for confirmatory Sanger sequencing of all variants presented in the study and deemed to be either pathogenic or putative ARly pathogenic (all chromosome positions are with reference to hg19 build).

**References**

1. McKenna A, Hanna M, Banks E, et al. The Genome Analysis Toolkit: a MapReduce framework for analyzing next-generation DNA sequencing data. Genome research 2010;20:1297-1303.

2. Van der Auwera GA CM, Hartl C, Poplin R, del Angel G, Levy-Moonshine A, Jordan T, Shakir K, Roazen D, Thibault J, Banks E, Garimella K, Altshuler D, Gabriel S, DePristo M,. From FastQ Data to High-Confidence Variant Calls: The Genome Analysis Toolkit Best Practices Pipeline. Current Protocols in Bioinformatics 2013;43:11.10.11-11.10.33.

3. Wang K, Li M, Hakonarson H. ANNOVAR: functional annotation of genetic variants from high-throughput sequencing data. Nucleic acids research 2010;38:e164.

4. Schwarz JM, Rodelsperger C, Schuelke M, Seelow D. MutationTaster evaluates disease-causing potential of sequence alterations. Nature methods 2010;7:575-576.

5. Kumar P, Henikoff S, Ng PC. Predicting the effects of coding non-synonymous variants on protein function using the SIFT algorithm. Nature protocols 2009;4:1073-1081.

6. Adzhubei IA, Schmidt S, Peshkin L, et al. A method and server for predicting damaging missense mutations. Nature methods 2010;7:248-249.

7. Chun S, Fay JC. Identification of deleterious mutations within three human genomes. Genome research 2009;19:1553-1561.

8. Richards CS, Bale S, Bellissimo DB, et al. ACMG recommendations for standards for interpretation and reporting of sequence variations: Revisions 2007. Genetics in medicine : official journal of the American College of Medical Genetics 2008;10:294-300.

9. Nemeth AH, Kwasniewska AC, Lise S, et al. Next generation sequencing for molecular diagnosis of neurological disorders using ataxias as a model. Brain 2013;136:3106-3118.

10. Service SCNLG. Neurogenetic Disorder Service NGS Panel [online]. Available at: <http://www.sheffieldchildrens.nhs.uk/downloads/labgenetics/LabGenetics_NGSNDTests.pdf>.
